# Supplementary material for: Biologically Potent Benzimidazole-Based-Substituted Benzaldehyde Derivatives as Potent Inhibitors for Alzheimer’s Disease along with Molecular Docking Study
Source: Pharmaceuticals (Basel). 2023 Jan 30;16(2):208. doi: 10.3390/ph16020208 (PMC9958709; doi:10.3390/ph16020208)
Supplement: Supplementary file 1 [file pharmaceuticals-16-00208-s001.zip › pharmaceuticals-2093252-SI.pdf]

Biologically Potent Benzimidazole-based-substituted benzaldehyde Derivatives as Potent Inhibitors for Alzheimer's Disease along with Molecular docking study

Bushra Adalat[1], Fazal Rahim[1], Wajid Rehman*[1], Zarshad Ali[1], Liaqat Rasheed[1], Yousaf Khan[2], Thoraya A. Farghaly[3], Sulaiman Shams[4], Muhammad Taha[5], Abdul Wadood[4], Syed A. A. Shah[6], Magda H. Abdellatif[7]

[1] Department of Chemistry, Hazara University, Mansehra 21300, Khyber Pakhtunkhwa, Pakistan
[2] Department of Chemistry, COMSATS University, Islamabad, Pakistan
[3] Department of Chemistry, Faculty of Applied Science, Umm Al-Qura University, Makkah, Almukkarramah, Saudi Arabia
[4] Department of Biochemistry, Abdul Wali Khan University Mardan, Mardan 23200, Pakistan
[5] Department of Clinical Pharmacy, Institute for Research and Medical Consultations (IRMC), Imam Abdul Rahman Bin Faisal University, P.O. Box 31441, Dammam, Saudi Arabia
[6] Faculty of Pharmacy, Universiti Teknologi MARA Cawangan Selangor Kampus Puncak Alam, Bandar, Puncak Alam, Selangor 42300, Malaysia,
[7] Department of Chemistry, College of Sciences, Taif University, P. O Box 11099, Taif 21944, Saudi Arabia
*To whom corresponding should be addressed
sono_waj@yahoo.com (Wajid Rehman), Department of Chemistry, Hazara University Mansehra 21120, Pakistan.

Supplementary Data

1.1.3. 2-(3,4-dichlorophenyl)-5-methoxy-1H-benzo[d]imidazole (3)

^{1}HNMR (500 MHz, DMSO-d_6): δ 11.28 (s, 1H, NH), 8.13 (d, J = 7.3 Hz, 1H, Ar-H), 8.06 (d, J = 6.8 Hz, 1H, Benzimidazole-H), 7.85 (s, 1H, Benzimidazole-H), 7.55 (d, J = 6.7 Hz, 1H, Benzimidazole-H), 7.22 (s, 1H, Ar-H), 7.02 (d, J = 7.6 Hz, 1H, Ar-H), 2.31 (s, 3H, -OCH_3), ^{13}CNMR (125 MHz, DMSO-d_6): δ 167.5, 162.5, 150.6, 149.9, 142.1, 141.0, 137.2, 133.4, 131.7, 125.4, 121.4, 116.8, 114.0, 44.7. HR-EIMS: m/z Calcd. for $C_{14}H_{10}Cl_2N_2O$ $[M]^+$ 292.0170; Found. 292.0167.

1.1.4. 5-methoxy-2-(2-nitrophenyl)-1H-benzo[d]imidazole (4)

^{1}HNMR (500 MHz, DMSO-d_6): δ 11.37 (s, 1H, NH), 8.20 (dd, J = 7.6, 2.2 Hz, 1H, Ar-H), 8.14 (d, J = 7.5 Hz, 1H, Benzimidazole-H), 7.89 (s, 1H, Benzimidazole-H), 7.63 (d, J = 6.8 Hz, 1H, Benzimidazole-H), 7.22-7.19 (m, 1H, Ar-H), 7.18-7.15 (m, 1H, Ar-H), 7.08 (dd, J = 7.1, 2.3 Hz, 1H, Ar-H), 2.42 (s, 3H, -OCH_3), ^{13}CNMR (125 MHz, DMSO-d_6): δ 169.5, 161.5, 150.6, 148.9, 144.1, 141.0, 138.6, 127.2, 124.7, 122.4, 119.4, 118.8, 114.0, 46.7. HR-EIMS: m/z Calcd. for $C_{14}H_{11}N_3O_3$ $[M]^+$ 269.0800; Found. 269.0796.

1.1.5. 5-methoxy-2-(naphthalen-2-yl)-1H-benzo[d]imidazole (5)

^{1}HNMR (500 MHz, DMSO-d_6): δ 11.21 (s, 1H, NH), 7.65 (d, J = 7.5 Hz, 1H, Ar-H), 7.59 (d, J = 7.56 Hz, 1H, Benzimidazole-H), 7.51 (s, 1H, Benzimidazole-H), 7.40 (d, J = 7.2 Hz, 1H, Benzimidazole-H), 7.10 (s, 1H, Ar-H), 7.02 (d, J = 7.3 Hz, 1H, Ar-H), 6.96 (d, J = 6.8Hz, 2H, Ar-H), 6.90 (d, J = 7.3Hz, 2H, Ar-H), 2.18 (s, 3H, -OCH_3), ^{13}CNMR (125 MHz, DMSO-d_6): δ 165.2, 158.4, 156.3, 151.5, 148.9, 145.4, 141.3, 140.4, 139.6, 137.8, 135.1, 131.7, 130.6, 122.3, 119.1, 114.9, 110.5, 43.2. HR-EIMS: m/z Calcd. for $C_{18}H_{14}N_2O$ $[M]^+$ 274.1106; Found. 269.0793.

1.1.6. 2-(2,4-dimethylphenyl)-5-methoxy-1H-benzo[d]imidazole (6)

^{1}HNMR (500 MHz, DMSO-d_6): δ 11.20 (s, 1H, NH), 8.08 (d, J = 7.8 Hz, 1H, Ar-H), 8.00 (d, J = 7.8 Hz, 1H, Benzimidazole-H), 7.70 (s, 1H, Benzimidazole-H), 7.46 (d, J = 7.7 Hz, 1H, Benzimidazole-H), 7.15 (s, 1H, Ar-H), 7.00 (d, J = 7.6 Hz, 1H, Ar-H), 2.25 (s, 3H, -OCH_3), 1.99 (s, 6H, CH_3), ^{13}CNMR (125 MHz, DMSO-d_6): δ 165.5, 158.5, 156.6, 151.9, 148.1, 140.0, 139.1, 135.2, 130.7, 122.4, 119.4, 114.8, 110.0, 43.7, 34.7, 34.2. HR-EIMS: m/z Calcd. for $C_{16}H_{16}N_2O$ $[M]^+$ 252.1263; Found. 269.0790.

1.1.7. 2-(4-(benzyloxy) phenyl)-5-methoxy-1H-benzo[d]imidazole (7)

^{1}HNMR (500 MHz, DMSO-d_6): δ 11.35 (s, 1H, NH), 7.86 (d, J = 7.0 Hz, 2H, Ar-H), 7.84 (d, J = 7.9 Hz, 1H, Benzimidazole-H), 7.81 (s, 1H, Benzimidazole-H), 7.74 (d, J = 8.1 Hz, 1H, Benzimidazole-H), 7.25 (d, J = 7.5 Hz, 2H, Ar-H), 7.21 (dd, J = 7.3, 1.8 Hz, 1H, Ar-H), 7.03 (t, J = 6.8 Hz, 1H, Ar-H), 7.01-6.95 (m, 1H, Ar-H), 2.22 (s, 3H, -OCH_3), 1.85 (s, 1H, OCH_2), ^{13}CNMR (125 MHz, DMSO-d_6): δ 167.4, 162.8, 160.3, 150.2, 149.5, 145.6, 142.9, 141.2, 140.0, 137.4, 136.1, 133.6, 131.4, 129.0, 125.7, 121.4, 119.8, 116.7, 114.5, 44.1, 25.2. HR-EIMS: m/z Calcd. for $C_{21}H_{18}N_2O_2$ $[M]^+$ 330.1368; Found. 330.1364.

1.1.8. 4-(5-methoxy-1H-benzo[d]-imidazol-2-yl)-N,N-dimethylaniline (8)

^{1}HNMR (500 MHz, DMSO-d_6): δ 11.28 (s, 1H, NH), 8.11 (d, J = 7.8 Hz, 2H, Ar-H), 8.08 (d, J = 7.3 Hz, 1H, Benzimidazole-H), 7.80 (s, 1H, Benzimidazole-H), 7.48 (d, J = 7.4 Hz, 1H, Benzimidazole-H), 7.38 (d, J = 7.1 Hz, 2H, Ar-H), 2.30 (s, 3H, -OCH_3), 1.80 (s, 6H, CH_3), ^{13}CNMR (125 MHz, DMSO-d_6): δ 167.4, 159.3, 147.7, 146.8, 142.3, 139.4, 123.9, 125.1, 122.6, 120.3, 117.2, 116.7, 112.9, 44.6, 21.9, 21.3. HR-EIMS: m/z Calcd. for $C_{16}H_{17}N_3O$ $[M]^+$ 267.1372; Found. 267.1369.

1.1.9. 2-methoxy-5-(5-methoxy-1H-benzo[d]-imidazol-2-yl) phenol (9)

^{1}HNMR (500 MHz, DMSO-d_6): δ 11.23 (s, 1H, NH), 9.45 (s, 1H, OH), 8.04 (d, *J* = 7.8 Hz, 1H, Ar-H), 7.95 (d, *J* = 7.9 Hz, 1H, Benzimidazole-H), 7.65 (s, 1H, Benzimidazole-H), 7.41 (d, *J* = 7.2 Hz, 1H, Benzimidazole-H), 7.11 (s, 1H, Ar-H), 7.06 (d, *J* = 7.3 Hz, 1H, Ar-H), 2.22 (s, 3H, -OCH_3), 2.04 (s, 3H, CH_3), ^{13}CNMR (125 MHz, DMSO-d_6): δ 166.5, 157.5, 153.6, 150.9, 149.1, 143.5, 137.0, 133.2, 128.7, 126.4, 121.4, 115.8, 111.0, 42.7, 39.6. HR-EIMS: m/z Calcd. for $C_{15}H_{14}N_2O_3$ $[M]^+$ 270.1004; Found. 270.1001.

1.1.10. 2,4-dichloro-6-(5-methoxy-1H-benzo[d]imidazol-2-yl)phenol (10)

^{1}HNMR (500 MHz, DMSO-d_6): δ 11.26 (s, 1H, NH), 9.49 (s, 1H, OH), 8.07 (d, *J* = 7.2 Hz, 1H, Ar-H), 7.98 (d, *J* = 7.9 Hz, 1H, Benzimidazole-H), 7.69 (s, 1H, Benzimidazole-H), 7.44 (d, *J* = 7.5 Hz, 1H, Benzimidazole-H), 7.15 (s, 1H, Ar-H), 2.24 (s, 3H, -OCH_3), ^{13}CNMR (125 MHz, DMSO-d_6): δ 170.5, 165.5, 161.6, 155.9, 150.1, 144.0, 142.7, 140.2, 139.7, 133.4, 127.4, 125.8, 120.0, 46.7. HR-EIMS: m/z Calcd. for $C_{14}H_{10}C_{12}N_2O_2$ $[M]^+$ 308.0119; Found. 308.0115.

1.1.11. 2-(2,4-dichlorophenyl)-5-methoxy-1H-benzo[d]imidazole (11)

^{1}HNMR (500 MHz, DMSO-d_6): δ 11.29 (s, 1H, NH), 8.14 (d, *J* = 7.5 Hz, 1H, Ar-H), 8.07 (d, *J* = 7.0 Hz, 1H, Benzimidazole-H), 7.86 (s, 1H, Benzimidazole-H), 7.57 (d, *J* = 7.7 Hz, 1H, Benzimidazole-H), 7.20 (s, 1H, Ar-H), 7.00 (d, *J* = 7.6 Hz, 1H, Ar-H), 2.32 (s, 3H, -OCH_3), ^{13}CNMR (125 MHz, DMSO-d_6): δ 167.9, 162.3, 150.1, 149.6, 142.0, 141.7, 137.8, 131.5, 129.2, 125.3, 121.1, 116.2, 114.6, 44.4. HR-EIMS: m/z Calcd. for $C_{14}H_{10}C_{12}N_2O_2$ $[M]^+$ 292.0170; Found. 292.0167.

1.1.12. 1,4-bis(5-methoxy-1H-benzo[d]imidazol-2-yl) benzene (12)

^{1}HNMR (500 MHz, DMSO-d_6): δ 11.35 (s, 2H, NH), 8.20 (d, *J* = 8.0 Hz, 2H, Ar-H), 8.16 (d, *J* = 8.3 Hz, 2H, Benzimidazole-H), 7.90 (s, 2H, Benzimidazole-H), 7.61 (d, *J* = 7.8 Hz, 2H, Benzimidazole-H), 7.47 (d, *J* = 7.5 Hz, 2H, Ar-H), 2.38 (s, 6H, -OCH_3), ^{13}CNMR (125 MHz, DMSO-d_6): δ 169.9, 169.5, 164.2, 162.0, 160.4, 160.0, 155.8, 155.3, 151.3, 151.1, 148.4, 148.2, 145.8, 145.1, 141.1, 141.0, 139.6, 139.2, 132.3, 132.0, 127.2, 127.0, 121.7, 121.5, 119.9, 119.4, 45.6, 45.3. HR-EIMS: m/z Calcd. for $C_{22}H_{18}N_4O_2$ $[M]^+$ 370.1430; Fond. 370.1436.

1.1.13. 2-(anthracen-9-yl)-5-methoxy-1H-benzo[d]imidazole (13)

^{1}HNMR (500 MHz, DMSO-d_6): δ 11.05 (s, 1H, NH), 7.42 (d, 1H, Ar-H), 7.38 (d, *J* = 7.4 Hz, 1H, Benzimidazole-H), 7.32 (s, 1H, Benzimidazole-H), 7.28 (d, *J* = 8.4 Hz, 1H, Benzimidazole-H), 7.22 (d, *J* = 7.2 Hz, 4H, Ar-H), 7.17 (d, *J* = 7.1 Hz, 4H, Ar-H), 2.20 (s, 3H, -OCH_3), ^{13}CNMR (125 MHz, DMSO-d_6): δ 160.4, 159.8, 155.3, 154.2, 151.5, 142.6, 141.9, 136.2, 133.0, 132.4, 130.1, 128.6, 127.4, 122.0, 120.7, 120.4, 119.5, 117.8, 114.7, 111.5, 110.6, 44.1. HR-EIMS: m/z Calcld. for $C_{22}H_{16}N_2O$ $[M]^+$ 324.1263; Found. 324.1260.

1.1.14. 1-(5-methoxy-1H-benzo[d]-imidazol-2-yl) naphthalen-2-ol (14)

^{1}HNMR (500 MHz, DMSO-d_6): δ 11.39 (s, 1H, NH), 9.70 (s, 1H, OH), 8.36 (d, J = 1.6 Hz, 1H, Ar-H), 8.20 (d, J = 7.3 Hz, 1H, Benzimidazole-H), 8.19 (d, J = 8.0 Hz, 1H, Ar-H), 7.90 (s, 1H, Benzimidazole-H), 7.19 (d, J = 7.4 Hz, 1H, Benzimidazole-H), 7.10 (d, J = 8.2 Hz, 2H, Ar-H), 7.06 (d, J = 7.8 Hz, 2H, Ar-H), 3.90 (s, 3H, -OCH_3), ^{13}CNMR (125 MHz, DMSO-d_6): δ 168.2, 148.1, 148.6, 146.5, 145.6, 141.9, 135.1, 135.0, 126.5, 124.4, 121.2, 121.0, 120.3, 119.5, 117.5, 114.2, 108.4, 55.8. HR-EIMS: m/z Calcd. for $C_{18}H_{14}N_2O_2$ $[M]^+$ 290.1055; Found. 290.1052.

1.1.15. 4-(5-methoxy-1H-benzo[d]-imidazol-2-yl) phenol (15)

^{1}HNMR (500 MHz, DMSO-d_6): δ 11.35 (s, 1H, NH), 9.66 (s, 1H, OH), 8.30 (d, J = 1.7 Hz, 2H, Ar-H), 8.17 (d, J = 7.0 Hz, 1H, Benzimidazole-H), 8.15 (d, J = 8.4 Hz, 2H, Ar-H), 7.85 (s, 1H, Benzimidazole-H), 7.14 (d, J = 6.6 Hz, 1H, Benzimidazole-H), 3.85 (s, 3H, -OCH_3), ^{13}CNMR (125 MHz, DMSO-d_6): δ 168.7, 148.5, 148.3, 146.6, 145.7, 141.3, 135.2, 135.8, 126.9, 124.0, 121.1, 121.2, 120.5, 55.6. HR-EIMS: m/z Calcd. for $C_{14}H_{12}N_2O_2$ $[M]^+$ 240.0899; Found. 240.0894.

1.1.16. 3-(5-methoxy-1H-benzo[d]imidazol-2-yl) phenol (16)

^{1}HNMR (500 MHz, DMSO-d_6): δ 11.38 (s, 1H, NH), 9.68 (s, 1H, OH), 8.33 (d, J = 1.9 Hz, 1H, Ar-H), 8.19 (d, J = 6.9 Hz, 1H, Benzimidazole-H), 8.17 (dd, J = 8.5, 1.9Hz, 1H, Ar-H), 7.88 (s, 1H, Benzimidazole-H), 7.26 (dd, J = 6.5, 1.5 Hz, 1H, Ar-H), 7.17 (d, J = 6.8 Hz, 1H, Benzimidazole-H), 6.97 (t, J = 6.3 Hz, 1H, Ar-H), 3.89 (s, 3H, -OCH_3), ^{13}CNMR (125 MHz, DMSO-d_6): δ 168.9, 148.2, 148.1, 146.1, 145.9, 141.5, 135.6, 135.0, 126.0, 124.8, 121.5, 121.0, 120.8, 56.0. HR-EIMS: m/z Calcd. for $C_{14}H_{12}N_2O_2$ $[M]^+$ 240.0899; Found. 240.0894.

1.1.17. 2-(5-bromo-2-methoxyphenyl)-5-methoxy-1H-benzo[d]-imidazole (17)

^{1}HNMR (500 MHz, DMSO-d_6): δ 11.15 (s, 1H, NH), 8.12 (d, J = 7.4 Hz, 1H, Ar-H), 8.02 (d, J = 7.3 Hz, 1H, Benzimidazole-H), 7.80 (s, 1H, Benzimidazole-H), 7.52 (d, J = 8.1 Hz, 1H, Benzimidazole-H), 7.17 (s, 1H, Ar-H), 7.09 (d, J = 7.1 Hz, 1H, Ar-H), 2.28 (s, 3H, -OCH_3), 2.15 (s, 1H, -OCH_3), ^{13}CNMR (125 MHz, DMSO-d_6): δ 167.9, 162.3, 150.1, 149.6, 142.0, 141.7, 137.8, 131.5, 129.2, 125.3, 121.1, 116.2, 114.6, 44.4, 29.5. HR-EIMS: m/z Calcd. for $C_{15}H_{13}BrN_2O_2$ $[M]^+$ 332.0160; Found. 332.0157.

1.1.18. 2-(4-ethoxy-3-methoxyphenyl)-5-methoxy-1H-benzo[d]imidazole (18)

^{1}HNMR (500 MHz, DMSO-d_6): δ 11.10 (s, 1H, NH), 7.95 (d, J = 7.2 Hz, 1H, Ar-H), 7.84 (d, J = 7.9 Hz, 1H, Benzimidazole-H), 7.79 (s, 1H, Benzimidazole-H), 7.67 (d, J = 8.3 Hz, 1H, Benzimidazole-H), 7.40 (s, 1H, Ar-H), 7.29 (d, J = 7.1 Hz, 1H, Ar-H), 7.16 (dd, J = 7.5, 1.9 Hz, 1H, Ar-H), 7.06 (t, J = 6.9 Hz, 1H, Ar-H), 7.03-6.98

(m, 1H, Ar-H), 2.28 (s, 3H, -OCH_3), 2.15 (s, 1H, OCH_3), ^{13}CNMR (125 MHz, DMSO-d_6): δ 167.9, 162.3, 160.6, 150.1, 149.6, 145.7, 142.0, 141.7, 140.2, 137.8, 136.0, 133.7, 131.5, 129.2, 125.3, 121.1, 119.2, 116.2, 114.6, 44.4, 29.5. HR-EIMS: m/z Calcd. for $C_{17}H_{18}N_2O_3$ $[M]^+$ 298.1317; Found. 298.1314.

1.1.19. 2-(2,4-dimethoxyphenyl)-5-methoxy-1H-benzo[d]imidazole (19)

^{1}HNMR (500 MHz, DMSO-d_6): δ 11.15 (s, 1H, NH), 8.01 (d, J = 6.8 Hz, 1H, Ar-H), 7.98 (d, J = 7.7 Hz, 1H, Benzimidazole-H), 7.64 (s, 1H, Benzimidazole-H), 7.49 (d, J = 7.4 Hz, 1H, Benzimidazole-H), 7.13 (s, 1H, Ar-H), 7.06 (d, J = 7.4 Hz, 1H, Ar-H), 2.23 (s, 3H, OCH_3), 2.16 (s, 6H, -OCH_3), ^{13}CNMR (125 MHz, DMSO-d_6): δ 165.0, 158.2, 156.3, 151.5, 148.0, 140.9, 139.4, 135.7, 130.3, 122.1, 119.6, 114.2, 110.9, 43.5, 34.3, 34.0. HR-EIMS: m/z Calcd. for $C_{16}H_{16}N_2O_3$ $[M]^+$ 284.1161; Found. 284.11656.

1.1.20. 2-(4-bromo-2,5-dimethoxyphenyl)-5-methoxy-1H-benzo[d]imidazole (20)

^{1}HNMR (500 MHz, DMSO-d_6): δ 11.08 (s, 1H, NH), 7.93 (s, 1H, Ar-H), 7.90 (d, J = 7.4 Hz, 1H, Benzimidazole-H), 7.62 (s, 1H, Benzimidazole-H), 7.52 (d, J = 7.6 Hz, 1H, Benzimidazole-H), 7.10 (s, 1H, Ar-H), 2.20 (s, 3H, OCH_3), 2.16 (s, 3H, OCH_3), 2.12 (s, 3H, -OCH_3), ^{13}CNMR (125 MHz, DMSO-d_6): δ 163.0, 159.2, 157.3, 154.5, 150.0, 145.9, 141.4, 138.7, 133.3, 125.1, 121.6, 119.2, 116.9, 42.5, 33.3, 32.0. HR-EIMS: m/z Calcd. for $C_{16}H_{15}BrN_2O_3$ $[M]^+$ 362.0266; Found. 362.0262.

1.1.21. 2,6-dimethoxy-4-(5-methoxy-1H-benzo[d]-imidazol-2-yl) phenol (21)

^{1}HNMR (500 MHz, DMSO-d_6): δ 11.31 (s, 1H, NH), 9.33 (s, 1H, OH), 8.20 (s, 1H, Ar-H), 8.09 (d, J = 7.7 Hz, 1H, Benzimidazole-H), 7.59 (s, 1H, Benzimidazole-H), 7.50 (d, J = 7.0 Hz, 1H, Benzimidazole-H), 7.40 (s, 1H, Ar-H), 2.50 (s, 3H, OCH_3), 2.30 (s, 3H, -OCH_3), 2.25 (s, 3H, -OCH_3), ^{13}CNMR (125 MHz, DMSO-d_6): δ 168.0, 165.2, 156.3, 153.5, 151.0, 150.9, 147.4, 141.7, 137.3, 132.1, 130.6, 124.2, 120.9, 45.5, 39.3, 37.0. HR-EIMS: m/z Calcd. for $C_{16}H_{16}N_2O_4$ $[M]^+$ 300.1110; Found. 300.1107.
